# Effects of cognitive behavioural therapy and bright light therapy for insomnia in youths with eveningness: study protocol for a randomised controlled trial

**DOI:** 10.1186/s13063-024-08090-0

**Published:** 2024-04-09

**Authors:** Shirley Xin Li, Forrest Tin Wai Cheung, Ngan Yin Chan, Joey Wing Yan Chan, Jihui Zhang, Albert Martin Li, Colin A. Espie, Michael Gradisar, Yun-Kwok Wing

**Affiliations:** 1 Department of Psychology, Sleep Research Clinic and Laboratory, The University of Hong Kong, Pokfulam, Hong Kong SAR, China; 2 The State Key Laboratory of Brain and Cognitive Sciences, The University of Hong Kong, Hong Kong SAR, China; 3 Li Chiu Kong Family Sleep Assessment Unit, Department of Psychiatry, Faculty of Medicine, The Chinese University of Hong Kong, Hong Kong SAR, China; 4 Center for Sleep and Circadian Medicine, The Affiliated Brain Hospital of Guangzhou Medicine University, Guangzhou, Guangdong China; 5 Department of Paediatrics, Faculty of Medicine, The Chinese University of Hong Kong, Hong Kong SAR, China; 6 Nuffield Department of Clinical Neurosciences and Sleep and Circadian Neuroscience Institute, University of Oxford, Oxford, UK; 7 WINK Sleep Pty Ltd, Adelaide, Australia; 8 Sleep Cycle AB, Gothenburg, Sweden

**Keywords:** Sleep, Insomnia, Adolescent, Chronotype, Eveningness, Cognitive behavioural therapy, Light, Youth

## Abstract

**Background:**

Insomnia and eveningness are common and often comorbid conditions in youths. While cognitive behavioural therapy for insomnia (CBT-I) has been suggested as a promising intervention, it remains unclear whether it is sufficient to also address circadian issues in youths. In addition, despite that light has been shown to be effective in phase-shifting one’s circadian rhythm, there has been limited data on the effects of bright light therapy and its combination with CBT-I on sleep and circadian outcomes in youths. The current protocol outlines a randomised controlled trial that examines the efficacy of CBT-I and CBT-I plus bright light therapy (BLT) in reducing insomnia severity, improving mood symptoms and daytime functioning (e.g. sleepiness, fatigue, cognitive function), and improving subjective and objective sleep and circadian measures compared to a waitlist control group.

**Methods:**

We will carry out a randomised controlled trial (RCT) with 150 youths aged 12–24 who meet the criteria of insomnia and eveningness. Participants will be randomised into one of three groups: CBT-I with bright light therapy, CBT-I with placebo light, and waitlist control. Six sessions of CBT-I will be delivered in a group format, while participants will be currently asked to use a portable light device for 30 min daily immediately after awakening throughout the intervention period for bright light therapy. The CBT-I with light therapy group will receive bright constant green light (506 lx) while the CBT-I with placebo light group will receive the modified light device with the LEDs emitting less than 10 lx. All participants will be assessed at baseline and post-treatment, while the two active treatment groups will be additionally followed up at 1 month and 6 months post-intervention. The primary outcome will be insomnia severity, as measured by the Insomnia Severity Index. Secondary outcomes include self-reported mood, circadian, daytime functioning, and quality of life measures, as well as sleep parameters derived from actigraphy and sleep diary and neurocognitive assessments. Objective measures of the circadian phase using dim-light melatonin onset assessment and sleep parameters using polysomnography will also be included as the secondary outcomes.

**Discussion:**

This study will be the first RCT to directly compare the effects of CBT-I and BLT in youths with insomnia and eveningness. Findings from the study will provide evidence to inform the clinical management of insomnia problems and eveningness in youths.

**Trial registration:**

ClinicalTrials.gov NCT04256915. Registered on 5 February 2020.

**Supplementary Information:**

The online version contains supplementary material available at 10.1186/s13063-024-08090-0.

## Introduction

### Background

#### Insomnia and eveningness in youth

Insomnia is the most common sleep complaint in youths, with a prevalence rate of 7.8–23.8% for insomnia disorder [1–6] and up to 40% for insomnia symptoms [2]. Youth insomnia tends to run a chronic course and persist into adulthood [7, 8]. Fernandez-Mendoza and colleagues followed a group of children (*n* = 502, median age of 9 at baseline) for 15 years and found that children with insomnia symptoms continued to have sleep problems in adolescence (~ 54%; median age, 16) and young adulthood (~ 62%; median age, 24), respectively [8]. If left untreated, insomnia may be associated with long-term adverse outcomes, such as an increased risk for physical and mental health problems [9], incidences of substance use [6], impaired daytime functioning, and increased school absenteeism [10].

Another significant change during adolescence is a gradual shift in circadian preference towards eveningness. This increased evening preference is intrinsically associated with pubertal development [11, 12], during which peak eveningness is reached at the end of adolescence [13]. Eveningness in youths is often associated with a constellation of adverse outcomes, including an increased risk for physical [14], mental [15–17], and sleep problems [18]. It is also frequently implicated in insomnia symptoms in adolescents, with those having an eveningness tendency having up to a 2.5-fold risk of insomnia problems. Existing research suggested that eveningness is a unique contributing factor to insomnia in adolescents, where a misaligned circadian rhythm potentially contributes to the aetiology of insomnia and implicates the phenomenology of insomnia, particularly with sleep-onset insomnia [19, 20]. Moreover, increasing research has demonstrated the potential synergistic effects of eveningness and insomnia on daytime sleepiness and mental health outcomes, such as depressive and anxiety symptoms. Previous studies showed that adolescents with both eveningness and insomnia were 1.3–4.9 times more likely (compared to insomnia alone) and 2.8–6.9 times more likely (compared to eveningness alone) to develop mental health problems [17, 21]. While both insomnia and eveningness are linked to a wide range of adverse outcomes in youths, the research on the treatment for this vulnerable group has been limited to date. The intricate relationship between insomnia and eveningness and their associated consequences underscore the need for concurrently addressing sleep and circadian issues in youths.

#### Treatment for youth insomnia

Although cognitive behavioural therapy for insomnia (CBT-I) is recommended as the first-line treatment for insomnia in adults with substantial empirical support [22, 23], research on the efficacy of CBT-I in adolescents has been limited. Åslund et al. (2018) conducted a meta-analysis on the effect of CBT-I in school-age children and adolescents and found only six RCTs (528 pooled samples, mean age 14.6 years) which showed a small-to-large magnitude of improvements in sleep parameters, specifically in sleep onset latency (SOL) [24]. Another meta-analysis by Blake et al. (2017) on adolescent cognitive behavioural sleep interventions included 9 trials (357 pooled samples, mean age 15.0 years) and found that cognitive and behavioural interventions improved not only various subjectively and objectively measured sleep parameters, but also daytime sleepiness, depression, and anxiety severity [25]. Despite some findings supporting the efficacy of CBT-I in youths, it was observed that up to 25–40% of young people treated with CBT-I showed minimal treatment response or failed to achieve a remission [26], suggesting the need to consider and/or integrate alternative strategies to enhance treatment outcomes.

The Transdiagnostic Sleep and Circadian Intervention (TranS-C) is a programme that combined conventional therapeutic components from CBT-I with additional circadian-related components to address both sleep and circadian disturbances [27]. A series of studies have provided support for the TranS-C programme as a promising intervention for improving sleep and advancing the circadian phase in youths [28–30]. Specifically, in a study comparing the efficacy of TranS-C versus psychoeducation in 176 youths aged 10 to 18, TranS-C was found to result in significantly greater improvements in self-reported sleep and circadian measures at post-intervention [28]. In addition to the cognitive-behavioural approach, the use of chronobiotics has gained increasing attention as a therapeutic approach in recent years. Light is the most potent zeitgeber that entrains the endogenous circadian clock to the surrounding environment. Under controlled laboratory conditions, light has been shown to be effective in shifting the human circadian rhythm and subsequently used as a therapeutic intervention [31, 32]. In adults, bright light therapy (BLT) has been shown to improve circadian outcomes, insomnia symptoms, and depressive symptoms [33, 34]. However, the evidence of BLT in the youth population remains relatively limited. A study conducted by Gradisar et al. (2011) in adolescents with delayed sleep phase disorder, a clinical sleep disorder reflecting the extreme end of eveningness, showed moderate to large effects of CBT-I with adjunctive BLT in reducing sleep onset latency (*d* = 0.65–1.13), advancing bedtime and rise time (*d* = 0.89–1.24), and improving daytime functioning (*d* = 0.75–0.79) [35]. Nonetheless, previous research utilising light therapy in adolescents was mainly conducted in either mixed clinical samples [36] or those with delayed sleep phase disorder [35, 37]. It remains unclear how to address circadian issues in the context of youth insomnia, specifically whether CBT-I alone is sufficient to tackle eveningness in youths diagnosed with insomnia or if an adjunctive circadian-focused intervention, i.e. bright light therapy, could additionally address the circadian components of youth insomnia, thereby enhancing the treatment outcomes.

### Study objectives

Given the gap in the current literature and the need to address circadian-related factors in youths with insomnia, this study aims to examine the efficacy of CBT-I and CBT-I plus bright light therapy in reducing insomnia severity in adolescents compared with a waitlist control. The secondary aim of the study is to examine the effects of CBT-I and CBT-I plus bright light therapy on mood symptoms and daytime functioning (e.g. sleepiness, fatigue, cognitive performance), as well as subjective and objective sleep and circadian measures.

The primary hypotheses for the trial are as follows:

1. Both CBT-I and CBT-I plus BLT will lead to an improvement in self-reported insomnia symptoms by the end of treatment as compared to the waitlist control.
2. CBT-I plus BLT will additionally lead to an advancement of chronotype and sleep onset by the end of treatment compared to the waitlist control.
3. Changes in insomnia will mediate the changes in mood and other daytime symptoms.

The secondary hypotheses are as follows:

1. CBT-I and CBT-I plus BLT will improve objectively measured sleep outcomes, mood symptoms, daytime functioning, and insomnia-related behaviours and cognitions by the end of treatment compared to the waitlist control.
2. The treatment effects of CBT-I and CBT-I plus BLT will be maintained over time at 1 month and 6 months after treatment.

## Methods

### Trial design and setting

This study is a randomised, assessor-blind, parallel-group, controlled trial with three arms. The trial will be conducted across two centres in Hong Kong: The Sleep Research Clinic and Laboratory at the Department of Psychology of the University of Hong Kong and the Department of Psychiatry at the Chinese University of Hong Kong. Youths who meet the study criteria will be randomly assigned to one of the three groups: CBT-I with adjunctive bright light therapy (CBTI + BLT), CBT-I with placebo light therapy (CBTI-BLT), or waitlist control (WL). The study protocol was approved by the Human Research Ethics Committee at The University of Hong Kong (Ref: EA1802063) and the Joint CUHK-NTEC Clinical Research Ethics Committee (Ref: 2019.339). The present protocol used the SPIRIT reporting guidelines [38] (The SPIRIT Checklist can be viewed in supplementary file 1).

### Participants

Participants aged 12–24 years will be recruited from the community, local schools, universities, and hospitals via social media promotion, flyers, mass emails, and invitation letters sent to all relevant institutions in Hong Kong. This age range was chosen to reflect the puberty onset and a wider span of adolescence till the early phase of young adulthood, where sleep and circadian issues continue to be prevalent [13]. The inclusion criteria are as follows: (1) having a chief complaint of difficulty initiating sleep at least three times a week for 3 months or more, with clinically significant impairment or distress, (2) scored ≥ 9 on the Insomnia Severity Index (ISI) [39], a suggested cut-off for clinical insomnia in youths [40], (3) scored ≤ 11 on the reduced Morningness-Eveningness Questionnaire (rMEQ) [41], the conventional cut-off for classifying evening type, (4) having a sleep onset time at or later than 23:15 (for those aged 12), 23:30 (for those aged 13–14), or 00:00 (for those aged 15 or above) for at least three nights per week, as confirmed by a prospective 7-day sleep diary. Participants will be excluded if they meet the following exclusion criteria: (1) having a current diagnosis or a history of manic or hypomanic episodes, schizophrenia spectrum disorders, neurodevelopmental disorders, organic mental disorders, intellectual disabilities, or substance abuse or dependence, (2) having a prominent medical condition known to interfere with sleep continuity and quality (e.g. eczema, gastro-oesophageal reflux disease), (3) having a clinically diagnosed sleep disorder that may disrupt sleep continuity and quality (e.g. narcolepsy, sleep-disordered breathing, restless leg syndrome) except for insomnia disorder and delayed sleep–wake phase disorder, (4) concurrent and regular use of medications known to affect sleep continuity and quality (e.g. hypnotics), (5) initiation or change of medications that could interfere with the circadian rhythm (e.g. lithium, melatonin supplements, melatonin agonist) within the past 3 months, (6) presence of clinically significant suicidal ideation with a plan or an attempt, (7) concurrently receiving any forms of structured psychotherapy, (8) presence of hearing or speech deficit or eye disease (e.g. retinal blindness, severe cataract), (9) being a night shift worker, and (10) had trans-meridian flight in the past 3 months or during the study.

### Randomisation and masking

Randomisation will be completed using simple randomisation with a 1:1:1 allocation ratio. A pre-randomised list with random sorting of the three trial arms will be generated in Microsoft Excel using the = RAND() function. As each participant enrols in the study, he/she will be sequentially assigned to the corresponding group in the pre-randomised list. The entire randomisation procedure will be completed by a trained research assistant who is not involved in other aspects of the trial. The project therapists and clinical assessors will be blinded to the group allocation. We do not anticipate any requirement for unblinding but if required, the Trial Manager, Data Coordinator, and Implementation Support Facilitators will have access to group allocations and any unblinding will be reported.

### Procedures

**Fig. 1:**
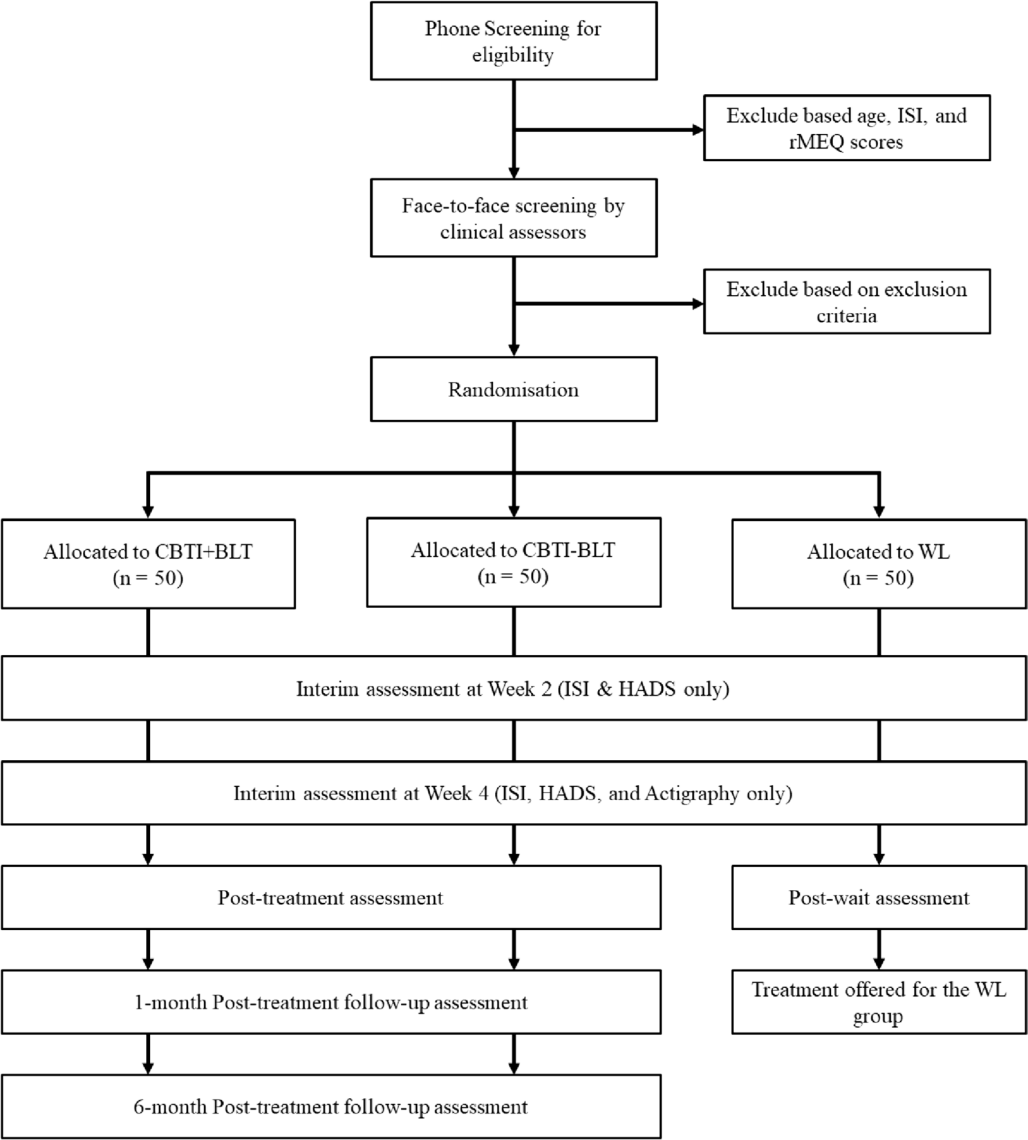
Flowchart of study design. CBTI + BLT cognitive behavioural therapy with active bright light therapy, CBTI-BLT cognitive behavioural therapy with placebo light therapy, HADS Hospital Anxiety and Depression Scale, ISI Insomnia Severity Index, rMEQ reduced Morningness-Eveningness Scale, WL waitlist control

Interested individuals can register for the study using an online application form and will be initially screened based on age, ISI score ≥ 9, and rMEQ score ≤ 11. Potential eligible individuals will then be invited to attend a face-to-face clinical interview to ascertain their eligibility for the trial. The diagnoses of sleep disorders and psychiatric disorders will be ascertained using the Diagnostic Interview for Sleep Patterns and Disorders [42] and the Mini International Neuropsychological Interview, respectively [43]. After confirming their eligibility, participants will be randomised to one of the following groups: CBTI + BLT, CBTI-BLT, and WL. Assessments are scheduled at pre-treatment (Week 0), during treatment (Week 2 and Week 4), and post-treatment (Week 7). Assessments at Week 2 and Week 4 will consist of two self-report measures (Insomnia Severity Index and Hospital Anxiety and Depression Scale) for monitoring the clinical status of the participants. Actigraphy will also be used in Week 4 to objectively monitor sleep and circadian-related changes and light exposure across the groups. The two active intervention arms (CBTI + BLT and CBTI-BLT) will be additionally followed up at 1 month and 6 months after the conclusion of the last group session. Participants in the WL group will be offered the treatment (either CBTI + BLT or CBTI-BLT) after completing the post-wait assessment. Figure 1 shows the study flowchart, and the study schedule is summarised in Table 1. Participants will receive HKD 500 (~ USD 64) at the end of the follow-up period as a token of appreciation of their participation in this trial.

**Table 1:** Study schedule

Timeline	Baseline			Intervention	Post-treatment		
	Enrolment	Pre-treatment assessment	Allocation	Treatment	Post-treatment assessment	1-month follow-up	6-month follow-up
Enrolment							
Screening for eligibility	●						
Informed consent	●						
Allocation			●				
Intervention							
CBTI + BLT		●		●	●	●	●
CBTI-BLT		●		●	●	●	●
Waitlist Control		●			●		
Data Collection							
Primary outcomes		●			●	●	●
ISI		●		●	●	●	●
Secondary outcomes		●			●	●	●
PSQI		●			●	●	●
MCTQ		●			●	●	●
CDRS		●			●	●	●
HADS		●		●	●	●	●
DSI-SS		●			●	●	●
PDSS		●			●	●	●
MFI		●			●	●	●
KIDSCREEN-27		●			●	●	●
Sleep diary parameters		●		●	●	●	●
Actigraphy parameters		●			●	●	●
PSG parameters		●			●		
DLMO assessment		●			●		
Neurocognitive assessments		●			●		●

*CSRS* the Children’s depression rating scale, *DLMO* Dim-light melatonin onset, *DSI-SS* Depressive symptoms inventory suicidality subscale, *HADS* Hospital anxiety and depression scale, *ISI* Insomnia severity index, *MCTQ* Munch chronotype questionnaire, *MFI* Multidimensional fatigue inventory, *PDSS* Paediatric daytime sleepiness scale, *PSQI* Pittsburgh sleep quality index

### Measures

#### Primary outcomes

The primary outcome is the change in insomnia symptoms as measured by the Insomnia Severity Index (ISI). The ISI is a seven-item self-rated scale designed to measure one’s insomnia problems, satisfaction with sleep, noticeability and worries towards one’s sleep problems, and insomnia-related interference with daily life. The possible score ranges from 0 to 28, where a higher score indicates greater insomnia severity. The ISI has been validated in local youths demonstrating good construct validity and high internal consistency with a Cronbach’s alpha of 0.83 [40].

#### Secondary outcomes

The secondary outcomes include changes in subjective sleep quality as measured by the Pittsburgh Sleep Quality Index (PSQI) [44], self-report chronotype as measured by the Munch Chronotype Questionnaire (MCTQ) [45], clinician-rated depressive symptoms as measured by the Children’s Depression Rating Scale (CDRS) [46], self-report mood symptoms as measured by the Hospital Anxiety and Depression Scale (HADS) [47], and suicidal ideation as measured by the Depressive Symptoms Inventory Suicidality Subscale (DSI-SS) [48]. Daytime functioning is subjectively assessed by the Paediatric Daytime Sleepiness Scale (PDSS) for daytime sleepiness and the Multidimensional Fatigue Inventory (MFI) for daytime fatigue [49, 50]. Secondary outcomes also include a change in the quality of life as measured by the KIDSCREEN-27 and the overall severity of clinical symptoms assessed by clinicians using the Clinical Global Impression Scale [51].

Other sleep-related outcomes will be evaluated subjectively using a sleep diary and objectively using actigraphy. Participants will be asked to complete a 7-day sleep diary while wearing a wrist actigraphy (Actiwatch Spectrum Plus, Philips Respironics). The outcomes to be analysed from the sleep diary and actigraphy are sleep onset time, wake time, total time in bed (TIB), total sleep time (TST), sleep onset latency (SOL), wake after sleep onset (WASO), and sleep efficiency (SE) across the recording period. Epoch-by-epoch actigraphy data will be extracted using cosinor and nonparametric analysis for circadian rhythm computation [52, 53]. Circadian parameters derived from cosinor analysis include the acrophase, amplitude, and MESOR, while circadian parameters derived from the nonparametric analysis include interdaily stability, intradaily variability, relative amplitude, M10 onset (10 h of maximal activity), and L5 onset (5 h of the least activity). Table 2 summarises the circadian parameters from the actigraphy computation.

**Table 2:** Circadian parameters derived from actigraphy circadian computations

Variables	Explanations
Cosinor analysis	
Acrophase	Time of maximum activity (peak). Express as clock time
Amplitude	Differences in distance from the peak to the nadir
MESOR	The mean activity level of the fitted curve
Non-parametric analysis	
L5/M10 onset	Time of onset of the five least active hours (presumed to represent sleep) and the ten most active hours (presumed to represent daytime activity). Express as clock time
Intradaily variability (IV)	An index ranging from 0–1 quantifies the degree of rhythm fragmentation. A higher value reflects greater fragmentation, reflecting more daytime napping or nocturnal awakening
Interdaily stability (IS)	An index ranging from 0–1 reflects the synchronisation of rhythm to the light–dark cycle. A higher value reflects better synchronisation
Relative amplitude (RA)	Normalised differences in distance from L5 to M10. A higher value reflects the greater robustness of the circadian rhythm

Participants will also be invited to complete a series of computerised neurocognitive tasks to assess objective cognitive performance. The list of computerised neurocognitive tasks is summarised in Table 3.

**Table 3:** List of neurocognitive tasks

Tasks	Cognitive domains
Balloon Analogue Risk Task	Risk-taking and decision making
Chinese Auditory Verbal Learning Task	Episodic memory
Digit Span (forward and backwards)	Working memory
N-back Task	Working memory
Go/No-Go Task	Inhibitory control
Trail Making Test	Visual attention and task switching/executive function
Wisconsin Card Sorting Test	Set shifting/executive function
Dot-probe Task	Attention bias

#### Other secondary measures

Due to limited funding resources, only half of the participants in the two treatment arms will be invited to complete a two-night in-laboratory assessment at baseline and post-treatment to objectively assess one’s sleep and circadian phase. Data collection will start after more than 50% of participants have been enrolled. The first night will serve as the adaptation night, and dim-light melatonin onset (DLMO) assessment will be conducted according to the previously published protocol [54]. In brief, participants will be asked to provide a total of 17 salivary samples in 30-min intervals starting 6 h before and continuing for 2 h after the habitual bedtime under a dim light condition with the ambient light level maintained below 10 lx. The salivary melatonin specimens will then be assayed using ultra-performance liquid chromatography-tandem mass spectrometry. The clock time of DLMO, calculated with linear interpolation when the melatonin concentration exceeded and remained above an absolute threshold of 12.9 pmol/L for at least two additional samples, will be used to reflect the circadian phase [55]. On the second laboratory night, participants will complete a level I polysomnography (PSG; Grael PSG Amplifiers and Recorders, Compumedics). Electrodes and sensors will be placed according to the American Academy of Sleep Medicine Manual for Scoring of Sleep and Associated Events [56]. PSG signals will be acquired and analysed using Compumedics Profusion SLEEP4 and stored securely at the Sleep Research Clinic and Laboratory at the University of Hong Kong. PSG recordings will then be scored by registered polysomnographic technologists who are blinded from the group allocations. Polysomnography-based sleep parameters that are considered as the secondary outcomes include total time in bed (PSG-TIB), total sleep time (PSG-TST), sleep onset latency (PSG-SOL), wake after sleep onset (PSG-WASO), and sleep efficiency (PSG-SE).

### Intervention

#### Cognitive behavioural therapy for insomnia

The CBT-I programme consists of six weekly sessions, 90 min each. The sessions will be conducted in a group format (four to six participants per group) and facilitated by trained therapists. The group sessions are structured and adopted from a well-established protocol for treating insomnia with additional emphasis on the effect of the circadian rhythm [57]. The programme aims to address the behavioural, cognitive, and physiological factors perpetuating insomnia. Table 4 summarises the session content of the CBT-I programme.

**Table 4:** Summary of the CBT-I programme

Session	Topics
1	• Programme overview • Psychoeducation on normal sleep, circadian rhythm, and insomnia • Introduction to 3Ps model of insomnia and case formulation • Introduction to the use of a sleep diary
2	• Review of sleep diary • Introduction to bright light therapy using Re-Timer • Behavioural strategies (sleep restrictions, stimulus control) • Sleep hygiene education • Setting sleep windows
3	• Review of sleep diary and use of Re-Timer and adjustment of sleep windows • Introduction to different treatment approaches for insomnia • Stress management and relaxation exercises • Constructive worry techniques
4	• Review of sleep diary and use of Re-Timer and adjustment of sleep windows • Cognitive restructuring Part I
5	• Review of sleep diary and use of Re-Timer and adjustment of sleep windows • Cognitive restructuring Part II
6	• Review of sleep diary and use of Re-Timer and adjustment of sleep windows • Review of the programme • Consolidating gains and relapse prevention

#### Bright light therapy

Participants in both intervention arms will be instructed to wear a portable light therapy device for 30 min daily immediately after awakening (Re-Timer; https://www.re-timer.com). Re-Timer is equipped with four light-emitting diodes (LEDs; two on each side) emitting a constant green light (per manufacturer’s manual, 500 nm dominant wavelength at 506 lx). The efficacy and safety of the Re-Timer have been reported by previous research [31, 58, 59]. The CBTI + BLT group receiving an active BLT treatment will be provided with the original Re-Timer, whereas the CBTI-BLT group receiving the placebo BLT treatment will be provided with a modified Re-Timer. The modified Re-Timer has red cellophane filters and red colour cardboard paper (250 g/m^2^) covering the LEDs, which turns the visual light spectrum towards red colour and blocks most of the light emitted from the LEDs (< 10 lx). This level of the light emitted from the modified Re-Timer is considered insufficient to have a phase-shifting effect on the circadian rhythm [60]. During each group session, the therapist will individually review and prescribe timing for light therapy for each participant based on their sleep diary completed in the previous week, aiming to advance one’s rise time and gradually optimise the sleep schedule. The daily sleep diary will be used to record participants’ usage of the Re-Timer to analyse adherence to BLT intervention.

#### Treatment integrity and quality assurance

All intervention sessions will be delivered by clinical psychologists or doctoral students who have received extensive training in behavioural sleep medicine and circadian science. All therapists will meet regularly during the study to discuss specific issues regarding the implementation of the trial protocol and to ensure the consistency of the implementation of the treatment protocol. All therapy sessions will be video recorded, and a subset of the recordings (20%) will be randomly selected to be reviewed and closely scrutinised by the blind judges based on a list of quality checkpoints.

#### Safety and adverse events

The likelihood of having serious adverse events during this trial is expected to be minimal. Both CBT-I and light therapy are generally well-tolerated. A systematic review of 99 studies on the randomised controlled trials of CBT-I showed no serious adverse events attributed to CBT-I [61]. Similarly, a review of 43 studies on bright light therapy showed no evidence of ocular damage due to light therapy [62]. Nonetheless, there have been reports of an increased risk of hypomania (risk ratio = 4.91, 95% CI 1.66 to 14.46), agitation, headache, blurred vision, and eye irritation in the use of bright light therapy [63]. As such, the HADS and an adverse effects checklist [64] will be used biweekly during treatment (i.e. Week 2 and Week 4) and follow-up assessment to monitor potential mood changes, the frequency of adverse events, and the related daytime impairment severity. In addition, the research clinician will evaluate any suicidal thoughts and behaviour, as well as non-suicidal self-injury, during the intervention period. Participants will be removed from the study and referred for open treatment in the case of serious adverse events (e.g. acute suicide risk or attempt).

#### Compliance, treatment satisfaction, and credibility

Treatment compliance and session evaluation will be assessed by the Treatment Satisfaction Scale (TSS) and Treatment Component Adherence Scale (TCAS), both of which have been used in previous trials [65]. The daily sleep diary will be used to examine the adherence to bright light therapy, where participants will be asked to record how long and at what times they used Re-Timers each day. The six-item Credibility-Expectancy Questionnaire (CEQ) will be used to assess treatment expectancy and credibility of the intervention [66].

### Statistical analysis

#### Sample size estimation

The target sample size was estimated based on the primary hypothesis using a priori power analysis. We expected a medium effect size (Hedges’ *g* = 0.30–0.47) based on the previous research [33]. Using *g* = 0.30 as a more conservative effect size, a total of 111 participants was estimated assuming a significance of 0.05 and an 80% power. Accounting for a 30% anticipated attrition rate, the final target sample size for the overall trial was 144 (*n* = 48 per arm).

#### Main analysis

The main analyses will be conducted following the intention-to-treat principle. Multilevel modelling (MLM) with restricted maximum likelihood estimation and an unstructured variance–covariance matrix will be used to analyse the main hypotheses on outcome measures. The use of MLM in analysing RCT data has the advantage of accounting for individual differences as a random effect [67–69]. MLM also has advantages over the traditional general linear model approach (e.g. mixed model repeated measures ANOVA) as it is unaffected by randomly missing data and can handle both time-dependent and independent covariates [67, 69]. Missing data for dependent variables will be assumed to be missing at random and accounted for by the maximum likelihood estimation. Two sets of MLMs will be conducted; one will include three treatment arms (CBTI + BLT vs CBTI-BLT vs WL) and two timepoints (pre-treatment vs post-treatment) as fixed effects; the second set will consist of two treatment arms (CBTI + BLT vs CBTI-BLT) and four timepoints (pre-treatment vs post-treatment vs 1-month follow-up vs 6-month follow-up) as fixed effects. The treatment × time interaction will also be included as a fixed effect on both MLMs and will be considered the treatment effect, while repeated measures will be nested within individual participants as random intercepts. A *p*-value of 0.05 represents statistical significance in all analyses. The *p*-values on outcome measures will be adjusted using the Benjamini–Hochberg procedure with a false discovery rate set as 20% to account for multiple comparisons.

#### Other analysis

For analysing the mediating effect of insomnia on the changes in mood and other daytime symptoms, the mediator (i.e. insomnia) will be added to the multilevel models for each of the primary and secondary outcomes (described above). An interim analysis is planned to be conducted when more than half of the participants have completed the 6-month follow-up assessment. The principal investigator (PI; SXL) is responsible for reviewing the interim analysis results for efficacy and futility purposes.

### Data management and dissemination

Except for the participant log, all data will be anonymised and stored on a password-protected server where accessibility will be limited to the PI and senior research members. Periodic backups will also be performed on a network-attached storage. Data containing personal identifiers (i.e. name and contact information) will be kept for up to 5 years after the first publication, after which the personal identifiers will be removed for long-term retention. To ensure the data quality, a detailed study protocol was designed to standardise the data collection processes. Any data requiring manual entry will be entered and checked by two individuals. The PI (SXL) and a doctoral student (FTWC) are responsible for the storage and analysis of the data. The study results and relevant analyses will be submitted to peer-reviewed journals and presented at international conferences. Data and materials related to the trial will be made available to academic investigators upon reasonable request.

### Monitoring and responsibilities

This is an investigator-initiated clinical trial. The funder did not play any role in the conceptualisation and design of the study and collection, analysis, and interpretation of data and in writing the manuscript. The trial steering team comprising the PI (SXL) and two site-investigators (NYC, FTWC) will oversee and monitor the entire study process and are responsible for providing supervision and organisational support to the implementation of the trial. The trial implementation team, comprising research assistants and clinical psychologists, will be responsible for the day-to-day support of the trial. In addition, the PI will have bi-weekly meetings with teams at both sites to address any issues during the trial. Due to the non-invasive, non-pharmacological, and low-risk nature of the intervention, a data monitoring committee will not be assembled for this trial. Instead, data monitoring will be conducted by the trial steering team, which will hold monthly meetings with the trial implementation team to review trial progress and adherence to the study protocol.

### Patient public involvement

There was no public and patient involvement in the design of the protocol.

## Discussion

Youth is a critical period characterised by an increased prevalence of insomnia and eveningness. Both conditions are associated with shared yet unique contributing factors and are linked to multiple adverse outcomes, especially impaired mental health. Despite the high prevalence and comorbidity, it remains unclear how to effectively address these sleep and circadian issues in youths. To the authors’ best knowledge, this study will be the first to directly compare the effects of cognitive behavioural therapy for insomnia (CBT-I) with and without adjunctive bright light therapy (BLT) in youths with insomnia and eveningness. The current study comprises a wide range of outcome variables, including self-report, clinician-rated, and objectively measured variables, enabling an in-depth analysis of the treatment effects. In addition, the current study includes an extended follow-up period (6 months), which enables an examination of whether treatment effects can be sustained over time. However, several limitations warrant attention. First, adherence to BLT treatment is solely monitored by the participant’s self-report on the sleep diary, which limits the objectivity when examining adherence to therapy. Secondly, the study is conducted on a homogeneous sample of the population, potentially weakening the generalisability of the findings.

Overall, the findings from this study will provide preliminary evidence to guide future clinical practice on using two potentially promising and safe therapeutic approaches in managing insomnia and eveningness in youths. The findings will also shed light on understanding the interplay between sleep and circadian factors in the context of youth mental health.

## Trial status

Protocol version 1, September 26, 2023. The trial protocol was registered on ClinicalTrials.gov (Identifier: NCT04256915) on February 5, 2020. In the event of any amendments to the study protocol, the PI will notify the research ethics committee at both centres and the funders in writing. The revised protocol will be sent to both centres for documentation. Additionally, the protocol will be updated in the clinical trial registry to ensure transparency and accurate reporting of the study procedures.

Data collection commenced on March 1, 2020, and is expected to be completed by the end of December 2023. Publishing of this protocol was delayed because of uncertainties related to the COVID-19 pandemic.

### Supplementary Information

**Supplementary Material 1.**

## Data Availability

The corresponding author holds access to the final trial dataset. Any data and materials related to the study can be provided upon reasonable request.

## Abbreviations

BLT: Bright light therapy
CBT-I: Cognitive behavioural therapy for insomnia
CDRS: Children’s depression rating scale
CEQ: Credibility-expectancy questionnaire
DLMO: Dim-light melatonin onset
DSI-SS: Depressive symptoms inventory suicidality subscale
HADS: Hospital anxiety and depression scale
ISI: Insomnia severity index
MCTQ: Munch chronotype questionnaire
MFI: Multidimensional fatigue inventory
MLM: Multilevel modelling
PDSS: Paediatric daytime sleepiness scale
PI: Principal investigator
PSG: Polysomnography
PSQI: Pittsburgh sleep quality index
RCT: Randomised controlled trial
rMEQ: Reduced morningness-eveningness questionnaire
SE: Sleep efficiency
SOL: Sleep onset latency
TCAS: Treatment component adherence scale
TIB: Total time in bed
TSS: Treatment satisfaction scale
TST: Total sleep time
WASO: Wake after sleep onset
WL: Waitlist control
